# Relationship among family environment, self-control, friendship quality, and adolescents’ smartphone addiction in South Korea: Findings from nationwide data

**DOI:** 10.1371/journal.pone.0190896

**Published:** 2018-02-05

**Authors:** Hye-Jin Kim, Jin-Young Min, Kyoung-Bok Min, Tae-Jin Lee, Seunghyun Yoo

**Affiliations:** 1 Department of Preventive Medicine, College of Medicine, Seoul National University, Daehak-ro, Jongno-gu, Seoul, Republic of Korea; 2 Institute of Health and Environment, Seoul National University, Seoul, Republic of Korea; 3 Graduate School of Public Health, Seoul National University, Seoul, Republic of Korea; Leibniz Institute for Prevention Research and Epidemiology BIPS, GERMANY

## Abstract

**Background:**

Many studies have examined the negative impact on smartphone addiction in adolescents. Recent concerns have focused on predictors of smartphone addiction. This study aimed to investigate the association of adolescents’ smartphone addiction with family environment (specifically, domestic violence and parental addiction). We further investigated whether self-control and friendship quality, as predictors of smartphone addiction, may reduce the observed risk.

**Methods:**

We used the 2013 national survey on internet usage and utilization data from the National Information Agency of Korea. Information on exposure and covariates included self-reported experience of domestic violence and parental addiction, sociodemographic variables, and other variables potentially related to smartphone addiction. Smartphone addiction was estimated using a smartphone addiction proneness scale, a standardized measure developed by national institutions in Korea.

**Results:**

Adolescents who had experienced domestic violence (OR = 1.74; 95% CI: 1.23–2.45) and parental addiction (OR = 2.01; 95% CI: 1.24–3.27) were found to be at an increased risk for smartphone addiction after controlling for all potential variables. Furthermore, on classifying adolescents according to their level of self-control and friendship quality the association between domestic violence and parental addiction, and smartphone addiction was found to be significant in the group with adolescents with lower levels of self-control (OR = 2.87; 95% CI: 1.68–4.90 and OR = 1.95; 95% CI: 1.34–2.83) and friendship quality (OR = 2.33; 95% CI: 1.41–3.85 and OR = 1.83; 95% CI: 1.26–2.64).

**Conclusion:**

Our findings suggest that family dysfunction was significantly associated with smartphone addiction. We also observed that self-control and friendship quality act as protective factors against adolescents’ smartphone addiction.

## Introduction

With advancing technologies being employed in mobile devices and internet-based applications, smartphone use has sharply increased in recent years [1]. Smartphones have become essential devices in daily life; however, their technological benefits engender adverse side effects when they are overused [2, 3]. For example, smartphone addiction has been associated with psychosocial disorders, including depression, social anxiety, impulsivity, and sleeping disorders [4–6], and physical problems, including musculoskeletal disorders, migraine headaches, pain in the wrists and neck, blurred vision, pinch strength, and hand function [7–11].

Although smartphone addiction is observed across all age groups, it tends to be more prevalent among adolescents as they find smartphones captivating. Adolescence, a developmental window and transition period between childhood and adulthood, entails tumultuous physical, psychological, and social changes [12]. Therefore, adolescents are more likely to demonstrate risk-taking and novelty- and sensation-seeking social interactions and play behaviors in response to life stresses or changes in the brain’s structure and function [12, 13]; thus, adolescents are particularly susceptible to developing addictions [13]. Excessive smartphone usage in adolescents could be indicative of such addictive behavior. Smartphones have become a popular means of mobile communication, and they provide a platform for internet-based applications among adolescents; furthermore, they a means of managing adolescents’ stress and help them cope with friends- and school-related problems [13–15]. However, recent studies have highlighted the negative psychosocial and physical effects of smartphone addiction [16]. Moreover, internet use through smartphones has been considered to cause problematic behaviors such as violence, influenced by game-playing and cyber-bulling through social network services (SNSs); furthermore, as the internet was also considered an easily accessible source of pornography for adolescents [17].

Substantial efforts have been dedicated to determining predictors for excessive smartphone use in adolescents by considering individual (psychological), family, peer, and other factors (i.e., depressive state, parenting style, friendship satisfaction, smartphone contents) [18–20]. Of these, adolescents’ family environment could be important in shaping adolescent behaviors regarding smartphone use. The family is the fundamental unit of the society, and most adolescents are born and raised in this unit; it is under its influence that they develop their personalities. Thus, when adolescents are a part of troubled families, it can affect the development of their problematic behaviors (i.e., illicit drug use, alcohol abuse, and risky sexual behavior) [21]. Indeed, greater family stress has been shown to be a significant predictor of problem behaviors in adolescents [22–25].

In the present study, we investigated whether dysfunctional family environments, such as those involving domestic violence or parental addiction (i.e., alcohol, gambling, and drugs), were associated with smartphone addiction in adolescents. Moreover, we demonstrated the protective factors of smartphone addiction in adolescents who have experienced domestic violence or parental addiction by classifying adolescents according to self-control and friendship quality.

## Materials and methods

The study protocol was approved by the Institutional Review Board of the Seoul National University Hospital (IRB number: E-1702-027-829). Informed consent was exempted by the committee.

### Data source and study population

We used the data from the 2013 national survey on Internet usage and utilization data from the National Information Agency (NIA) of Korea. This survey data has been collected annually since 2004 to promote the policy for prevention and resolution of internet and smartphone addiction. Since 2006, it has been designated as including nationally approved statistics and has been utilized in research. The 2013 survey on Internet usage and utilization includes questions related to daily internet use, usage by the type of service, awareness of internet addiction, smartphone use, and psychosocial characteristics. This data is a population-based, cross-sectional survey designed to collect community level national epidemiological data for population between 5 and 54 years of age. Sampling was allocated based on age, gender, and 17 regions. A skilled investigator visited each individual’s household, and the health information of the subjects was collected through face-to-face interviews with the investigator. A total of 17,500 people participated in the 2013 survey. Of these, this study included 3380 adolescents excluding those not enrolled in school or those without smartphones.

### Domestic violence and parental addiction

Information regarding the respondents’ experience with domestic violence and parental addiction was obtained from self-reports. Domestic violence was determined by a question asking whether the participant had experienced it. Parental addiction included respondents who had experienced parental drug problems, problematic alcohol use, and/or gambling problems. The responses to the above two question were coded as dichotomous variables.

### Smartphone addiction

Smartphone addiction is defined as a state in which a person is immersed in smartphone usage and cannot control themselves [26, 27]. Smartphone addiction was measured on the smartphone addiction proneness scale developed by NIA in 2011. Smartphone addiction was measured on the smartphone addiction proneness scale developed by the NIA in 2011 [28]. This scale was developed by adding items reflecting the unique nature of smartphone to internet addiction scale [29], and had a high internal consistency with Cronbach's α of .880, making it suitable for screening those at risk for smartphone addiction (28). The scale comprised four subcomponents consisting of tolerance (4 questions), withdrawal (4 questions), virtual life orientation (5 questions), disturbance of adaptive functions (2 questions). These were rated on a four-point Likert scale (1 = not at all and 4 = always). According to the self-diagnostic criteria for the youth, smartphone addiction is classified as follows: a high-risk group (total score, ≤45; disturbance of adaptive functions, ≤16; withdrawal score, ≤13; and tolerance score, ≤14), potential-risk group (total score, 42–44; disturbance of adaptive functions, ≤14; withdrawal score, ≤12; and tolerance score, ≤13) and a general-user group (not belonging to either of the other groups). Finally, based on the smartphone addiction risk group classification criteria, the high-risk and the potential-risk groups were included in the smartphone over use group.

### Other variables

Other variables of interest included demographic and family characteristics, such as school age, sex, monthly income and residence; family structure including single-parent family or two-parent family; parents’ economic activities including dual-income family or single-income; the level of academic achievements; and the experience of preventive education for smartphone addiction. The level of self-control was determined by the following: “whether the respondent has the potential to overcome crisis or difficulty quickly” and “whether the respondent has a sense of control”. These rated on a four-point Likert scale. We also asked questions to determine the level of friendship quality: “whether the respondent is satisfied with relationships with people online” and “whether the respondent is satisfied with relationships with people offline”. If respondents of each two questions had ratings of 7–8, we classified them into the “high” level; if they had ratings of 2–6, we classified them into the “not high” level.

### Statistical analysis

The chi-square test was used to evaluate the differences in smartphone addiction according to participant characteristics. Unadjusted and multivariate-adjusted logistic regression analyses were performed to demonstrate the association between domestic violence and addicted parents and adolescents’ smartphone addiction. The odds ratio (OR) with the corresponding 95% confidence interval (95%CI) for the smartphone addiction were generated by the experience of domestic violence or addicted parents. The association resulting from the multivariate-adjusted model assessing the relation between domestic violence and addicted parents and adolescents’ smartphone addiction was adjusted for school age, sex, monthly income, single-parent family, double-income family, academic achievement, and preventive education for smartphone addiction. We also categorized the level of peer relationship satisfaction and self-control as either “high” or “not high”. Furthermore, we calculated the OR and 95%CI for smartphone addiction by the rate of domestic violence and addicted parents and classified it according to peer relationship satisfaction and self-control (either “high” or “not high”). All statistical analyses were performed using AS 9.2 software (SAS Institute, Cary, NC, USA), and statistical significance was evaluated at a significance level of 0.05 (α < 0.05).

## Results

Table 1 showed the characteristics of the study population by smartphone addiction. We identified the percentage of participants who were middle school aged adolescents (*p* = 0.0031), in two parent families (*p* = 0.0212), or in double-income families (*p* = 0.0212); these adolescents were more likely to addicted to smartphones. However, there were no significant differences in terms of sex, monthly income, or residence.

**Table 1:** Characteristics of the study population by smartphone addiction, n(%).

	Smartphone addiction				
	Yes (n = 881)		No (n = 2,569)		*p*-value
School age					
Elementary school age (10-13yr)	239	(29.47)	847	(32.97)	0.0031
Middle school age (14-16yr)	352	(43.40)	944	(36.75)	
High school age(17-19yr)	220	(27.13)	778	(30.28)	
Sex					
Male	419	(51.66)	1314	(51.15)	0.7976
Female	392	(48.34)	1255	(48.85)	
Monthly income (1000 KRW)					
≤ 2,000,000	79	(9.74)	270	(10.51)	0.6469
2,000,000–4,000,000	418	(51.54)	1346	(52.39)	
≥4,000,000	314	(38.72)	953	(37.10)	
Residence					
Metropolis	353	(43.53)	1181	(45.97)	0.3905
Small and medium city	390	(48.09)	1165	(45.35)	
Rural areas	68	(8.38)	223	(8.68)	
Family structure					
Single-parent	83	(10.23)	277	(10.78)	0.6591
Two-parents	728	(89.77)	2292	(89.22)	
Parents’ economic activity					
Dual-earners	508	(62.64)	1492	(58.08)	0.0212
Single-earners	303	(37.36)	1077	(41.92)	
Academic achievement					
Low	319	(39.33)	989	(38.50)	0.6697
High	492	(60.67)	1580	(61.50)	
Preventive education for smartphone addiction					
Yes	226	(27.87)	716	(38.5)	0.9983
No	585	(72.13)	1853	(61.50)	

**Fig 1:**
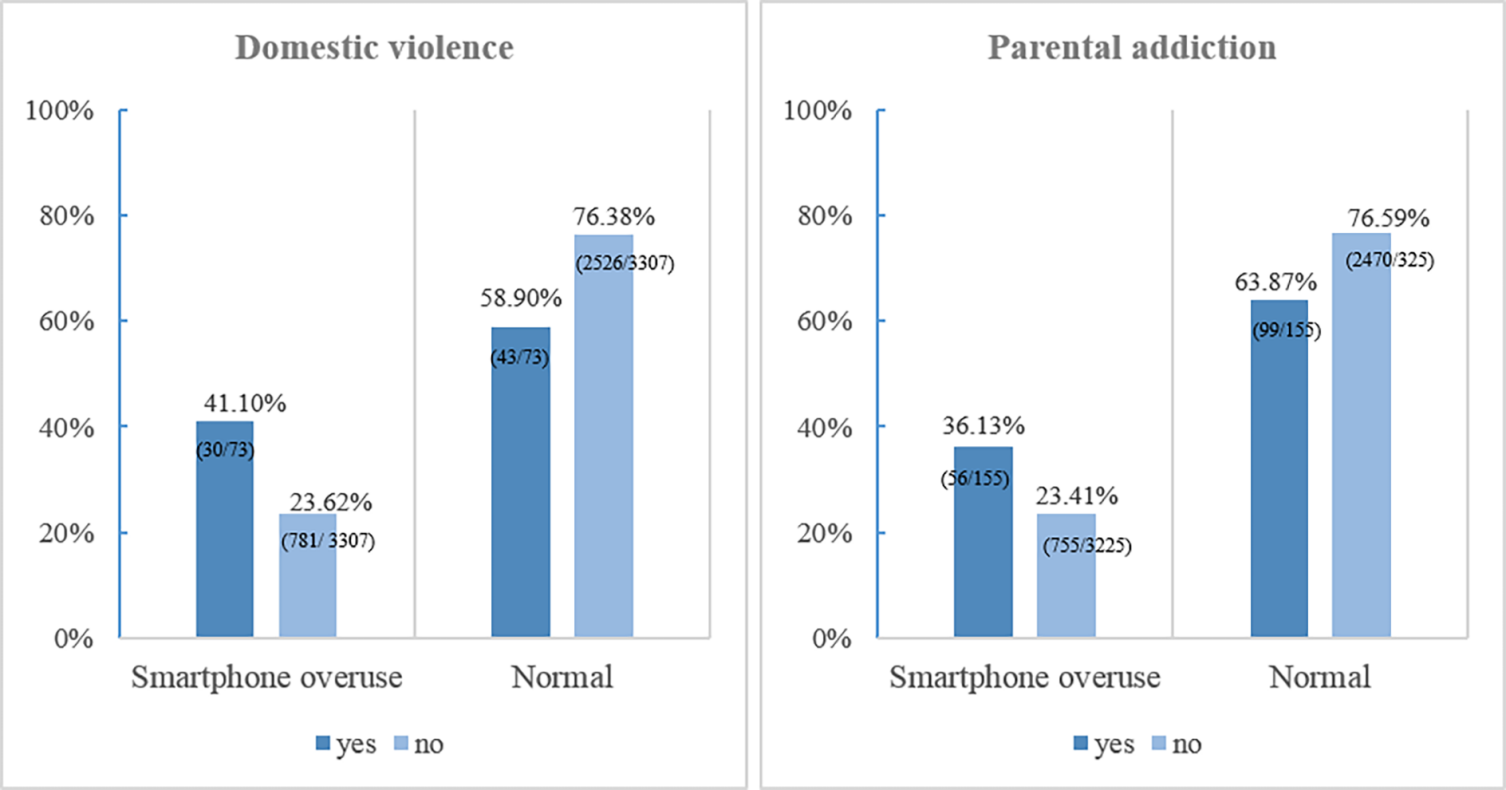
Percentage (%) and number (case/total) of family dysfunction (domestic violence and parental addiction) by adolescents’ smartphone addiction.

Fig 1 displayed the Percentage (%) and number (case/total) of family dysfunction (domestic violence and parental addiction) by adolescents’ smartphone addiction. The percentage of adolescents with domestic violence (”yes”) was higher than those without domestic violence (”no”) in smartphone addiction group (41.10% vs. 23.62%). However, the percentage of adolescents with domestic violence (”yes”) was lower than their counterparts in normal group (58.90% vs. 76.38%). In the same manner, the percentage of parental addiction “yes” group (36.13%) was higher than “no” group (23.41%) in Smartphone addiction group. On the contrary, the percentage of parental addiction “no” group (63.87%) is higher than “yes” group (76.59%) in smartphone addiction group.

Table 2 showed the OR (95%CI) of smartphone addiction according to domestic violence and addicted parents. We implemented a series of ordered logistic regression analyses. Compared with the unadjusted model, although each adjusted OR for domestic violence decreased, it remained significant after adjustments were made for age, sex, income, and residence (model 1) and on further controlling family structure, parents’ economic activity, academic achievement, preventive education for smartphone addiction (model 2). We also found a significant association between domestic violence and addicted parents and smartphone addiction after adjusting for demographic variables (model 1) and family structure, parents’ economic activity, academic achievement, preventive education for smartphone addiction (model 2).

**Table 2:** Odds ratio (95% CI) for smartphone addiction by family dysfunction (domestic violence and parental addiction).

	Unadjusted model		Adjusted model*	
Domestic violence				
Yes	1.71	(1.22–2.42)	1.74	(1.23–2.45)
No	Reference		Reference	
Parental addiction				
Yes	2.01	(1.24–3.25)	2.01	(1.24–3.27)
No	Reference		Reference	

* Adjusted model was adjusted for school age, sex, income, and residence family structure, parents’ economic activity, academic achievement, preventive education for smartphone addiction

**Fig 2:**
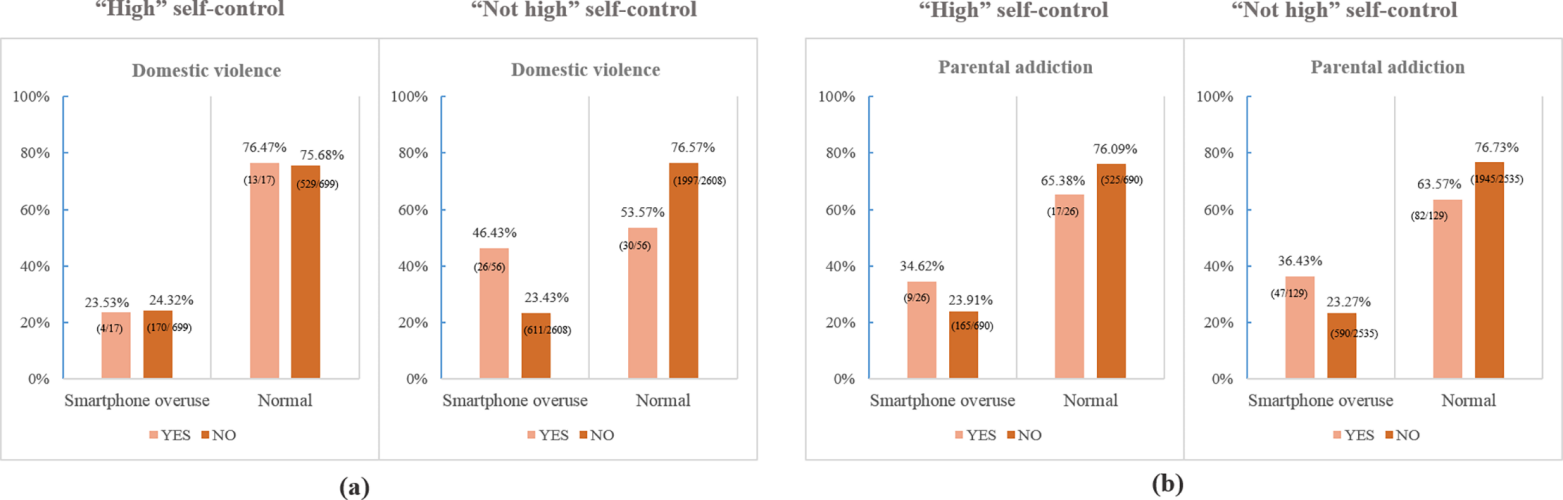
Percentage (%) and number (case/total) of family dysfunction (domestic violence and parental addiction) by adolescents’ smartphone addiction depending on their self-control level.

Fig 2 displayed the Percentage (%) and number (case/total) of family dysfunction (domestic violence and parental addiction) by adolescents’ smartphone addiction depending on their self-control level. Regarding “high” level of self-control in Fig 2A, there was little differences in the experience of domestic violence (“yes” or “no”) between smartphone addiction group and normal group. On the other hands, percentage of adolescents with domestic violence (”yes”) was higher than their counterparts (“no”) in smartphone addiction group (46.43%). Otherwise, opposite results was identified in Normal group in Fig 2B. The percentage of adolescents with addicted parents was higher in Smartphone addiction group than in Normal group regardless of self-control level, whereas the percentage of parental addiction in Smartphone addiction group was lower in “high” level of self-control (34.62%) than”not high” level (36.43%).

**Fig 3:**
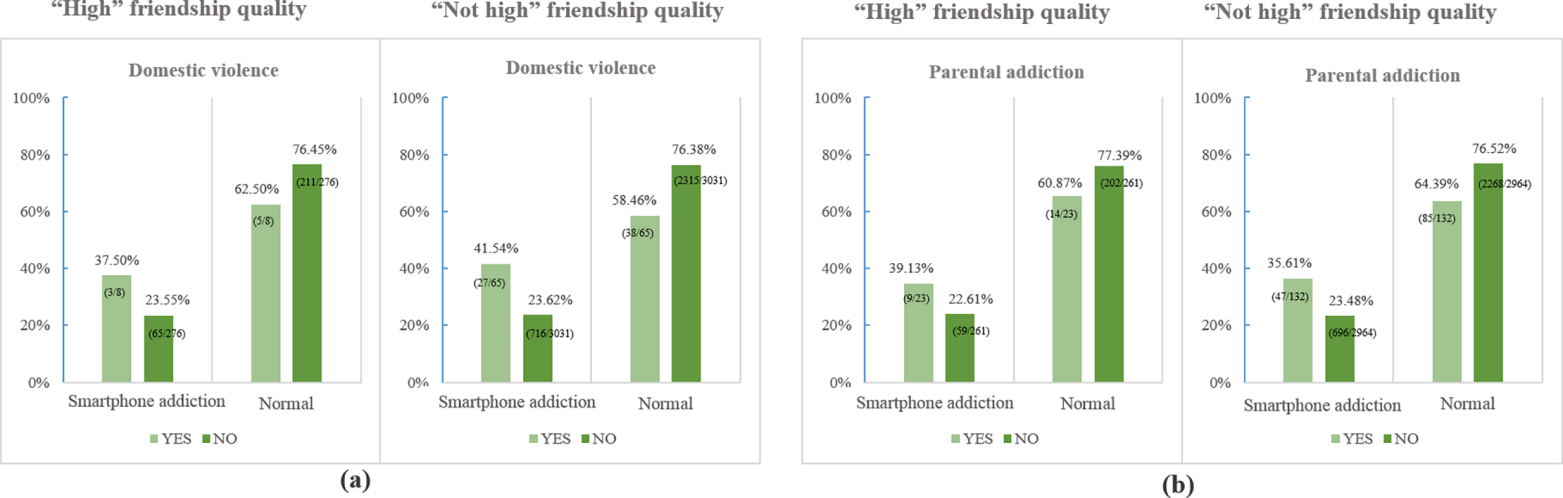
Percentage (%) and number (case/total) of family dysfunction (domestic violence and parental addiction) by adolescents’ smartphone addiction depending on their friendship quality level.

Fig 3 displayed the Percentage (%) and number (case/total) of family dysfunction (domestic violence and parental addiction) by adolescents’ smartphone addiction depending on their friendship quality. The percentage of adolescents with domestic violence was higher in smartphone addiction group than in normal group, regardless of friendship quality (Fig 3A). On the contrary, the percentage of domestic violence of smartphone addiction group was lower in “high” level of self-control (37.50%) than “not high” level (41.54%). In addition, the percentage of parental addiction of smartphone addiction group was higher in “high” level of friendship quality (39.13%) than “not high” friendship quality (35.61%).

Table 3 showed the OR (95% CI) for smartphone addiction against the experience of domestic violence and exposure to parents’ addiction problem depending on the level of friendship quality (“high” vs “not high”) controlled for demographic variables (school age, sex, income, and residence) and other variables potentially related to smartphone addiction (family structure, parents’ economic activity, academic achievement, and preventive education for smartphone addiction). After classifying participants according to the level of friendship quality (“high” vs “not high”), those who had experienced domestic violence were found more likely to be addicted to smartphones if they reported “not high” friendship quality. Respondents with addicted parents were similarly more likely to be addicted to smartphones if they had reported “not high” friendship quality. In particular, the OR for experience of domestic violence for smartphone addiction was 2.33 (95% CI: 1.41–3.85), while that for having problem related to addicted parents was 1.83 (95% CI: 1.26–2.64) in group with “not high” friendship quality. However, in the case of “high” friendship quality, no significant association was observed between domestic violence and addicted parents and smartphone addiction.

**Table 3:** Odds ratio (95% CI) for smartphone addiction by domestic violence and parental addiction (“high” vs “not high” friendship quality).

	“high” friendship quality ^a^		“not high” friendship quality ^a^		
	Odds ratio (95% CI)		Odds ratio (95% CI)		
Domestic violence					
Yes	1.80	(0.38–8.51)	2.33		(1.41–3.85)
No	Reference		Reference		
Parental addiction					
Yes	2.33	(0.90–6.05)	1.83	(1.26–2.64)	
No	Reference		Reference		

^a^Adjusted by school age, sex, income, residence family structure, parents’ economic activity, academic achievement, preventive education for smartphone addiction education.

Table 4 showed the OR (95% CI) for smartphone addiction contrasted with the experience of domestic violence and exposure to parents’ addiction problem according to the level of self-control control (“high” vs “not high”), which was controlled for demographic variables (school age, sex, income, and residence) and other variables potentially related to smartphone addiction (family structure, parents’ economic activity, academic achievement, and preventive education for smartphone addiction). A similar pattern was identified in Tables 3 and 4. After participants were classified according to their level of self-control (“high” vs “not high”), those with domestic violence and “not high” levels of self-control were found to be addicted to their smartphone. Participants with addicted parents and “not high” levels of self-control were similarly more likely to be addicted to their smartphones. To be specific, the OR for experience of domestic violence for smartphone addiction was 2.87 (95% CI: 1.68–4.90) and the OR for exposure to parents’ addiction problem was 1.95 (95% CI, 1.34–2.83) the in group with “not high” self-control. However, for adolescents with “high” levels of self-control (HIGH), no significant relation was observed between domestic violence and addicted parents and smartphone addiction.

**Table 4:** Odds ratio (95% CI) for smartphone addiction by domestic violence and parental addiction (“high” vs “not high” Self-control).

	“high” Self-control ^a^		“not high” Self-control ^a^	
	Odds ratio (95% CI)		Odds ratio (95% CI)	
Domestic violence				
Yes	0.95	(0.30–3.01)	2.87	(1.68–4.90)
No	Reference		Reference	
Parental addiction				
Yes	1.79	(0.77–4.17)	1.95	(1.34–2.83)
No	Reference		Reference	

^a^Adjusted by school age, sex, income, residence family structure, parents’ economic activity, academic achievement, preventive education for smartphone addiction education.

## Discussion

We found that family dysfunction in terms of domestic violence and parental addiction was significantly associated with adolescent smartphone addiction in South Korea. On classifying adolescents depending on whether they had “high” or “not high” self-control and friendship quality, we found that the observed association between family dysfunction and smartphone addiction was not significant in adolescents with “high” self-control or friendship quality. Thus, adolescents exposed to domestic violence or with addicted parents had an increased likelihood of smartphone addiction, but having “high” self-control or friendship quality could protect against it.

To the best of our knowledge, no evidence exists to indicate an association between family dysfunction and smartphone addiction in adolescents. However, based on prior findings on the significant association between dysfunctional families and adolescents’ problematic behaviors [30, 31], smartphone addiction could also be affected by family dysfunction. The negative aspects of the family environment are presumably key risk factors for smartphone addiction.

In particular, poor family relationships due to violence or child abuse showed a high risk of being manifested as anti-social behaviors, poor attention span, hyperactivity [32–34], and violent behaviors, including substance abuse and other forms of delinquency [35, 36]. Internalized symptoms (i.e., depression, aggressiveness, low level of self-esteem, and anxiety) caused by domestic violence was significantly associated with adolescents’ addiction to the internet and games [37, 38]. Moreover, parents’ addition to alcohol, drugs, or gambling negatively impacted physical and behavioral problems among adolescents. For instance, alcoholism, constituting the largest proportion of parental addiction in our study, is often accompanied by abuse, domestic violence, and mental symptoms and can lead to anxiety and anger among adolescents [39]. Children with alcoholic parents showed social maladjustments such as interpersonal conflicts, flight, aggression, and drinking behavior [40]. Admittedly, in the current study, we cannot clearly define the role of the family environment in terms of smartphone addiction in adolescents. Considering that smartphone addiction is a newly emerging problem in adolescent behavior [41], the observed association in our study could be understood from these previous, related findings on family dysfunction with adolescents’ problem behaviors.

Of course, removing the dysfunctional factors of the family (domestic violence and parental addiction) would be a good solution for mitigating the risk of smartphone addiction among adolescents. However, as family issues cannot be resolved or changed by a single individual, it is also important to find a protective factor to prevent negative events that induce smartphone addiction. In the present study, we considered self-control and friendship quality as protective factors against risky adolescent behavior, specifically smartphone addiction. We defined self-control as an individual’s ability to control his/her emotions, thoughts, and behaviors against impulses and temptations [42]. Historically, self-control has often been used as a general theory to explain individual differences in criminality [43]. Studies have demonstrated the link between low self-control and delinquency [44], imprudent behaviors [45, 46], and more recently, smartphone addiction [47]. As smartphones have special characteristics of immediacy and portability, they are available anytime and anywhere; thus, self-control has been identified as the important feature required for controlling smartphone addiction in adolescents [48].

Another important protective factor with respect to smartphone addiction is the quality of peer relationships. Positive interaction with peers and sustained social intimacy can buffer psychosocial problems such as anxiety and depression in adolescents [49], despite negative stress in their families. Furthermore, positive peer relationships can provide social support, sense of stability, and recognition from others that cannot be obtained from parents [50]. However, adolescents who feel isolated from peer relationships in the virtual world are at risk of engaging in communication and seeking self-esteem or reassurance through online games and SNS [51, 52]. Overall, adolescents with negative family stresses are more likely to be smartphone over-users. Nevertheless, those who have high self-control and friendship quality are less likely to be addicted to smartphones.

To the best our knowledge, this is the first study that investigates the relation between adolescents’ smartphone addiction and family environments, especially in terms of domestic violence and parental addiction, using the nationwide data from South Korea. However, several limitations should be considered. First, although we have analyzed the national survey data, the self-reported items, “self-control” and “friendship quality”, have not been verified for reliability and validity. However, the questions on self-control and friendship quality are consistent with the previously stated meaning and definition. For example, self-control refers to the ability to regulate one’s actions and thoughts independently of one’s own situation [53]. The questions included adolescents’ activation of self-control in response to overcome crisis or difficulty quickly and their sense of self-control. They showed relatively high internal consistency, with a Cronbach’s α of .70. In addition, the friendship quality was based on online and offline relationship satisfaction [54]. Adolescents’ online and offline relationships represent the comprehensive relationships with peers, and relationship satisfaction refers to relationship quality among them [54, 55]. Second, this study had a cross-sectional design; thus, the causal relations between exposure and outcomes cannot be directly ascertained. Third, other variables not considered in the study models may have a confounding effect. In particular, it is impossible to include personal psychological factors, peer and school factors, smartphone device characteristics, and parents’ educational background and job information, which are significant in the research of adolescents’ problematic behaviors and development. Fourth, specific data on the type and intensity of domestic violence is unavailable; thus, the experience of domestic violence can be underestimated. Furthermore, estimates of parental alcohol, gambling, and drug addiction also have limitations in terms of definite interpretation because cases of slight deviation or serious addiction could not be distinguished. Finally, it is expected that smartphone addiction will vary between individuals in terms of content.

In conclusion, the current study investigated how adolescents experienced domestic violence and how those with addicted parents have a higher likelihood of smartphone addiction. Furthermore, even if adolescents belong to dysfunctional families, higher levels of self-control and peer relationship satisfaction has been shown to decrease the risk for smartphone addiction. Although the results of this study need to be further clarified and validated by further studies, our study provides evidence regarding the potential effect of family stress on increasing the risk of smartphone addiction and also identified a solution to partially alleviate the risk.
